# Increased Blue Mood and Loneliness during COVID-19 Pandemic in a Veteran Cohort

**DOI:** 10.1093/geroni/igaa057.3523

**Published:** 2020-12-16

**Authors:** Nora Mattek, Rachel Wall, Zachary Beattie, Chao-Yi Wu, Jeffrey Kaye, Hiroko Dodge, Lisa Silbert

**Affiliations:** 1 Oregon Health & Science University, Portland, Oregon, United States; 2 Portland Veterans Affairs Medical Center, Portland, Oregon, United States; 3 Layton Alzheimer’s Disease Center, Portland, Oregon, United States

## Abstract

Older Veterans are at especially high risk of depression and social isolation due to COVID-19 stay-at-home orders and necessary safety precautions. We aimed to objectively measure differences in mood reports before and after COVID-19 stay-at-home orders in rural older Veterans. Participants age > 62 were enrolled in the Collaborative Aging Research using Technology (CART) initiative, a NIH and VA HSRD funded multi-site study examining the feasibility of unobtrusive remote sensing and monitoring of physical, cognitive, and health-related activities. The VA CART site consists of Pacific Northwest Veteran volunteers and their cohabitants. Weekly online health forms including questions about blue mood and loneliness were collected January – July 2020. A COVID stay-at-home order was instituted March 13 2020. Generalized estimating equations (GEE) with logit link was used to investigate differences in mood reports pre- and post- stay-at-home orders. 100 older volunteers completed 2441 health reports (mean age 71.2 years, 41% female, 19% single). Thirty-five percent were urban, 34% large rural, and 31% small rural using rural-urban commuting area (RUCA) scores urban: 1-3; large rural 4-6; small rural 7-10. After adjusting for covariates, incidence of blue mood and loneliness reports were significantly higher after stay-at-home orders (OR=4.4, p<0.0001 and OR=7.2, p<0.0001 respectively). Results varied by rurality with large rural volunteers showing the largest increases. Real-world monitoring of weekly health reports may identify those at greatest risk for depression and social isolation in older Veterans and their cohabitants and is of particular relevance in rural settings, where access to specialty care is limited.

